# Health-related quality of life assessment in head and neck cancer: A systematic review of phase II and III clinical trials

**DOI:** 10.1016/j.heliyon.2024.e40671

**Published:** 2024-11-24

**Authors:** Daria Maria Filippini, Francesca Carosi, Olimpia Panepinto, Giacomo Neri, Elisabetta Nobili, Nastassja Tober, Raffaele Giusti, Massimo Di Maio

**Affiliations:** a Medical Oncology, IRCCS Azienda Ospedaliero-Universitaria di Bologna, Italy; b Department of Oncology, University of Turin, AOU Città della Salute e della Scienza di Torino, Turin, Italy; c Division of Oncology, Department of Translational Medicine, University of Piemonte Orientale, Novara, Italy; d Azienda Ospedaliera Maggiore Della Caritá, Novara, Italy; e Medical Oncology Unit, Sant’Andrea Hospital of Rome, Italy

**Keywords:** Head and neck cancer, Quality of life, Clinical trials, Endpoint

## Abstract

**Background:**

Patients with head and neck cancer (HNC) bear a significant load, due to both disease-related symptoms and to toxicities associated with treatments. Evaluating quality of life (QoL) is crucial to gauge the physical and psychological impact on these patients. Our primary aim was to assess whether QoL has been incorporated as an endpoint in phase II and III clinical trials for HNC patients in the last 15 years.

**Material and methods:**

We investigated publications from 11 major journals to identify randomized and non-randomized phase II and phase III clinical trials assessing locoregional and systemic treatments as either single or multimodal strategies, published from 2008 to March 2023 in patients with HNC.

**Results:**

We screened 2045 studies and we selected 158 articles that met the eligibility criteria including a total of 31.734 patients. Globally, QoL was the primary end point in 2 publications (1 %), secondary in 38 (24 %), and exploratory in 7 (4 %). The quota of primary publications with QoL among endpoints increased over time: 14 (17 %) publications between 2008 and 2015 and 33 (42 %) between 2016 and 2023. Notably, in phase III trials, QoL was included among endpoints in 30 (49 %) publications, whereas in phase II studies, QoL was present in 17 (17 %).

**Conclusions:**

In HNC, the assessment of QoL as an endpoint in clinical trials is still missing, even in phase III trials. Efforts should be focused on the adoption of Patient-Reported Outcomes (PROs) in trials to improve the definition of treatment value in this vulnerable population.

## 1 Introduction

### 1.1 Head and neck cancer landscape and the burden of toxicities

Head and neck cancer (HNC) is the seventh most common cancer worldwide, with 900.000 new diagnoses and 450.000 deaths each year [1]. Head and neck squamous cell cancer (HNSCC) represents more than 90 % of cases and the rest includes rare and heterogenous histotypes. Tobacco and alcohol consumption are the main risk factors for HNC, causing around 75–85 % of these cancers followed by Human Papillomavirus (HPV) infection that is involved in the etiopathogenesis of oropharyngeal cancers [2].

The current standard of care for locally advanced HNC encompasses a combination of surgery, radiotherapy (RT) and chemotherapy (CT), with the latter two often administered concurrently as chemoradiotherapy (CRT) while immune checkpoint inhibitors (ICIs) have been introduced for the recurrent/metastatic setting. Despite the evolution of treatment modalities, such as transoral robotic surgery, advancements in radiotherapy techniques including intensity-modulated radiotherapy (IMRT) and the introduction of ICIs, these interventions are still associated with both acute and long-term side effects, often manifesting as severe events that exert a substantial impact on the quality of life (QoL) outcomes.

First, the pain management in patients with HNC is challenging not only due to the specific disease localization and various contributing factors such as inflammation, edema, ulceration, nerves infiltration, infection but also to the impact of treatments: half of the patients reported pain before treatment and 81 % during treatment; moreover, the pain remains uncontrolled after 6 months from the primary treatment in 36 % of patients [3,4].

Second, locoregional treatments enhance the impairments in the functional capacity of the upper aerodigestive tract: swallowing, speech, and respiratory functions are often altered in patients with HNC, worsened by anatomical disruption caused by surgery and RT, with a long-life impact.

Indeed, while acute toxicities associated with RT, such as mucositis, xerostomia, and edema, typically show substantial improvement in most of patients upon completion of active treatments, the late toxicities, including neuropathy and fibrosis of the oral, laryngeal, and pharyngeal musculature, may manifest years after the end of therapy.

It has been demonstrated that within the initial year post-RT, approximately half of HNC survivors experience dysphagia [5,6] and dysarthria [7], with the persistence and exacerbation of these conditions observed even after 10-year post-RT [8,9]. Dysphagia may initially arise from factors such as tumor size and localization, with subsequent contributions from RT-related pain, mucositis, and xerostomia often exacerbating the condition, leading to weight loss and a decline in nutritional status both during and after treatment [10]. A weight loss exceeding 5 % during treatment is common in HNC patients, observed in 66 % of cases [11,12]. This critical weight loss often leads to malnutrition, a condition commonly associated with treatment interruptions and unfavorable prognosis [13,14].

Furthermore, enteral nutrition is often required in patients with HNC, either with a nasogastric tube (NGT) or a percutaneous endoscopic gastrostomy (PEG), impairing long-term dysphagia and PEG dependency due to atrophy of the muscles involved in swallowing function. This results in a poor QoL due to the interference with daily activities and social eating [15,16].

Even if less common in modern series (less than 5 %), radiotherapy-associated osteoradionecrosis represents an impactful complication of HNC treatments; however, advances in preventive oral and dental care, along with the development of three-dimensional radiotherapy and IMRT, have significantly reduced its incidence [17].

An additional burden is represented by the systemic effects related to CT, including fatigue, higher risk of infection, hematological toxicities, nausea and vomiting, sore or dry mouth, numbness or tingling in hands or feet, hearing impairment, tinnitus, and hair thinning. Not surprisingly, patients with HNC are particularly vulnerable to psycho-social issues, undergoing significant changes in their appearance and physical well-being. Even after the end of treatment, patients may continue to experience anxiety, isolation and depression, especially if premorbid depression was present, with a demonstrated negative clinical impact. In fact, patients with HNC suffering from depression have shown a worse compliance with medical treatment recommendations and greater depressive symptoms were associated with a higher mortality [18,19]. Since the treatment of HNC must rely on a multidisciplinary approach, it is mandatory to care about HNC patients' rehabilitation not only to improve their functional outcomes but also to improve their mental health and QoL.

### 1.2 Quality of life assessment in head and neck cancer

In head and neck oncology, QoL assessment is a fundamental goal, but only in 2009 the Food and Drug Administration (FDA) finalized a guidance to include patient-reported outcomes (PROs) in clinical trials, having the capability to identify symptoms and toxicities which may be underestimated by clinicians but significantly impact QoL [20].

The World Health Organization (WHO) defines QoL as "an individuals’ perception of their position in life within the context of the culture and value systems they live in, and in relation to their goals, expectations, standards, and concerns" [21]. Over the years, numerous definitions of QoL have emerged, incorporating both subjective and objective factors. Consequently, statements about QoL differ based on its definition and the assessment methods used in the literature.

Several specific tools designed for QoL assessment in HNC are readily available, as outlined in Table 1. Among these, the EORTC Core Quality of Life questionnaire (EORTC QLQ-C30) is one of the most widely utilized tools: utilizing 30 items, it encompasses functional, symptomatic, and global health and QoL scales [22].

**Table 1:** Specific scales for QoL assessment in patients with HNC.

Name of tool	Physical domain	Psycho-social domain	Other items
QLQ-H&N35 (supplementary module to QLQ-C30) [23]	Pain, swallowing, taste/smell, speech	Social eating, social contact, sexuality	6 single symptoms (problems with teeth, opening mouth, dry mouth, sticky saliva, coughing, feeling ill)
FACT-H&N [24]	Physical well-being	Social-family well-being, Emotional well-being, Functional well-being	12 single symptoms (dry mouth, breathing, voice quality, eating quality, swallowing, alcohol consumption, smoking habit, communication issues, locoregional pain, appearance)
University of Washington Questionnaire (UW-QOL) [25]	Pain, swallowing, chewing, speech, shoulder dysfunction, taste, production of saliva	Appearance, recreation, activity, mood, anxiety	3 general questions concerning overall QoL individual perception
MD Anderson Symptom Inventory -Head and Neck (MDASI-HN) [26]	Pain, Fatigue, Nausea, Disturbed sleep, Distress/feeling upset, Shortness of breath, Difficulty remembering, Lack of appetite, Drowsiness, Dry mouth, Sadness, Vomiting, Numbness/tingling	Activity, Working (including housework), Relations with other people, Enjoyment of life, Mood	9 specific symptoms: mucus in the mouth and throat, difficulty swallowing or chewing, choking or coughing, difficulty with voice or speech, skin pain/burning/rash, constipation, problems with tasting food, mouth/throat sores, problems with teeth or gums
University of Michigan Head and Neck Specific Quality of Life Instrument (HNQoL) [27]	Pain, feeding	Communication, emotions	

In 2014, Heutte et al. classified and rated ninety QoL scales aiming to constitute a list of validated instruments to use in head and neck oncology, adapted to different settings [28].

However, despite the increasing interest in QoL assessment for patients with HNC and the development of QoL instruments, there is no standard method of QoL assessment in HNC.

Considering that locally advanced HNC patients may live decades with the sequelae of their disease and treatment and that the clinical efficacy of a treatment does not always correspond to the optimal QoL result for patients, QoL assessment and monitoring during and after treatments is a compelling need in clinical trials to achieve a balance between risk and benefit.

The aim of this systematic review was to determine whether QoL is being considered among endpoints in phase II and phase III clinical trials conducted in patients with HNC during the last 15 years.

## 2 Materials and methods

This systematic review was undertaken and reported in accordance with the Preferred Reporting Items for Systematic Reviews and Meta-Analyses (PRISMA) guidelines [29].

### 2.1 Eligibility criteria

Existing literature was systematically searched to identify randomized and non-randomized phase II and phase III clinical trials evaluating both locoregional treatments of surgery and radiotherapy and systemic treatments at all sites and stages of HNC.

The inclusion criteria for this review were as follows: (1) original research (primary phase II and phase III clinical trials); (2) published in English; (3) focused on HNC (including oropharynx, hypopharynx, oral cavity, larynx, salivary glands cancers, nasopharynx); (4) interventional studies encompassing surgery, radiotherapy, chemotherapy, immunotherapy, targeted therapies, or a combination of them; (5) published in 11 main international journals (JAMA Oncology; JAMA; Journal of Clinical Oncology; The New England Journal of Medicine; The Lancet; The Lancet Oncology; Annals of Oncology; Cancer; The British journal of cancer; European Journal of Cancer; Journal of the National Cancer Institute); (6) publications from January 2008 to March 2023.

Trials that present results on QoL assessment in updates or secondary publications (even if not published in the 11 main international journals previously analyzed) are accurately categorized as "having a QoL endpoint."

The exclusion criteria were: (1) phase I clinical trials, preclinical studies, retrospective studies, review articles, systematic reviews, meta-analyses, commentaries, case reports, case series, and editorials; (2) publications centered on supportive treatments lacking direct anticancer efficacy, specifically those focused on symptom palliation through symptom-targeted interventions (e.g. for mucositis).

### 2.2 Search strategy

The authors interrogated the electronic database MEDLINE-PubMed using the search string - (((((((((((((((RANDOMISED) AND TRIAL) AND ("2008"[Date - Create]: "2023"[Date - Create])) AND "JAMA"[Journal]) OR "Lancet (London, England)"[Journal]) OR "The New England journal of medicine"[Journal]) OR "Annals of oncology: official journal of the European Society for Medical Oncology"[Journal]) OR "British journal of cancer"[Journal]) OR "Cancer"[Journal]) OR "JAMA Oncology"[Journal]) OR "European journal of cancer (Oxford, England: 1990)"[Journal]) OR "Journal of clinical oncology: official journal of the American Society of Clinical Oncology"[Journal]) OR "Journal of the National Cancer Institute"[Journal]) OR "The Lancet. Oncology"[Journal]) AND head and neck) AND cancer - selecting articles published from 2008 until 06/03/2023. The authors specifically selected publications available in 11 main journals.

Titles and abstracts were independently screened for relevance by four authors of this study (CF; FDM; NG; PO; TN) and cross checked by the first authors (CF/FDM), while disagreements were resolved by consensus-based discussion.

Data according to the study endpoints (primary/secondary/exploratory) were taken from the methods section of the paper and from the study protocol, when presented as supplementary material.

### 2.3 Data extraction

The authors extracted data using a standardized data sheet in Microsoft Excel, creating an electronic database. Data extraction tables were compiled to record study features and patient characteristics, classified by the following items: first authors, date of definitive and ahead-of-print publication, primary manuscript journal, open label versus blinded study, countries involved in the study, research design, sample size, control group, type of treatment, tumor site, QoL assessment.

Experimental treatments were classified into five main groups: chemotherapy; targeted agents; immunotherapy; surgery; radiotherapy as single modality treatment. In the chemotherapy group, the association with radiotherapy was allowed. Treatments were also subdivided according to the therapeutic intent: induction setting, adjuvant, exclusive, or first, second, third line of treatments, and other settings.

Research investigations were categorized into two groups, namely "negative" and "positive," based on the outcome of the primary endpoint.

The results of the five independent reviewers were compared, and disagreements on search strategy, article inclusion, and data extraction were resolved by discussion with two authors (FDM and CF).

## 3 Results

### 3.1 Study characteristics overview

The search identified 2045 publications, of which 368 were initially removed (guidelines, editorials, commentaries, erratum, and retracted publications). Among the 1677 publications, 828 records were excluded, due to non-inherent topics. The remaining 849 articles were deemed eligible according to the title and abstract of which 691 were subsequently excluded because of phase I clinical trials, pre-clinical studies, case reports, case series, retrospective studies, reviews and systematic reviews, meta-analysis, trials updates and secondary analysis.

**Fig. 1:**
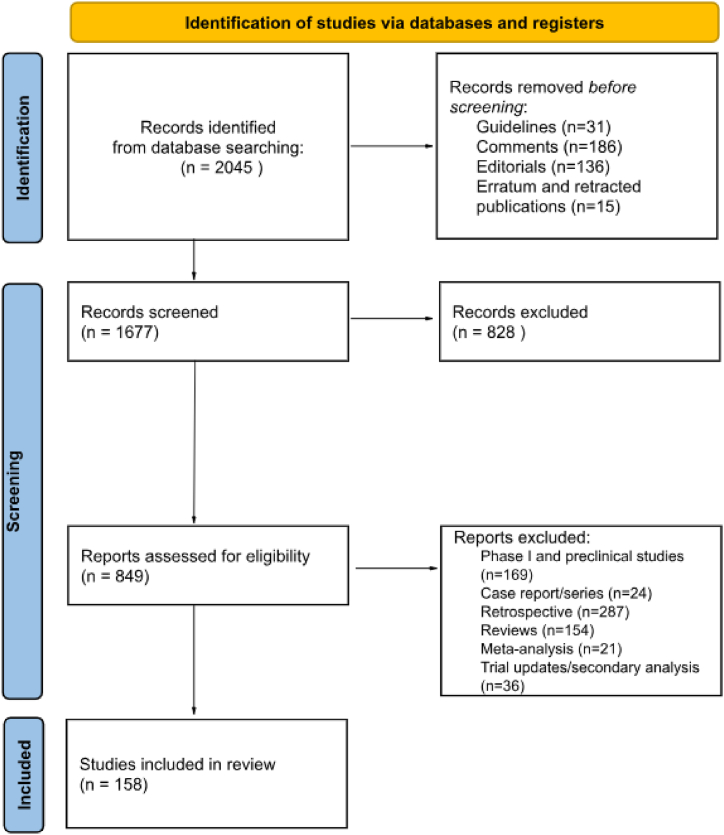
PRISMA 2020 flow diagram for new systematic reviews.

A flow diagram following the PRISMA 2020 guidelines illustrates the methodological steps involved in the screening and selection of articles (Fig. 1). Finally, 158 studies met the eligibility criteria and were included in the review, for a total of 31.734 patients. The main characteristics of the eligible publications are summarized in Table 2. Half of the studies (n = 79, 50 %) were published between 2008 and 2015, and the others were published between 2016 and 2023.

**Table 2:** Characteristics of 158 publications included.

Characteristic	N. of publications	% of publications
**Year of primary publication**		
2008	11	6.9 %
2009	1	0.6 %
2010	13	8.2 %
2011	12	7.6 %
2012	7	4.4 %
2013	13	8.2 %
2014	10	6.3 %
2015	12	7.6 %
2016	7	4.4 %
2017	12	7.6 %
2018	13	8.2 %
2019	14	9 %
2020	9	5.7 %
2021	10	6.3 %
2022	14	9 %
2023	0	0 %
**Journal Impact Factor**		
Low (<15)	41	25.9 %
High (>30)	117	74.1 %
**Funding**		
Profit	101	64 %
Non-profit	57	36 %
**Study design**		
Phase II	97	61.4 %
Phase III	61	38.6 %
**Masking**		
Open label	141	89.2 %
Blinded	17	10.8 %
**Type of experimental therapy**		
RT ± other (chemotherapy, target therapy)	59	37.4 %
Immunotherapy alone or in combination	18	11.4 %
Chemotherapy alone (first, second, third line)	23	14.5 %
Target therapy	52	33 %
Others (surgery, proton therapy)	6	3.7 %
**Treatment setting**		
Neoadjuvant/Induction	26	16.4 %
Adjuvant	12	7.6 %
Exclusive	39	24.7 %
Recurrent/metastatic	78	49.4 %
Others	3	1.9 %
**Main primary endpoint**		
OS	36	22.8 %
PFS	32	20.2 %
ORR	54	34.2 %
LRC	13	8.2 %
Others^a^	23	14.6 %
**Study results according to the primary endpoint**		
Positive	92	58.2 %
Negative	66	41.8 %

OS: overall survival; PFS: progression free survival; RR: response rate; ORR: overall response rate; LRC: locoregional control.

a Including the two trials with QoL as primary endpoint.

The majority of studies (n = 126, 79.7 %) encompassed patients with various localizations of HNSCC while the others focused on a specific subsite: 6 (3.8 %) centered on oropharynx, 6 (3.8 %) on oral cavity, 4 (2.5 %) on larynx, 14 (8.9 %) on salivary gland cancers and 2 (1.3 %) on nasopharynx. Among the included articles, 61 (38.6 %) were phase III and 97 (61.4 %) were phase II studies. Concerning the masking design, the majority of the studies (n = 141, 89.2 %) employed an open-label approach, while seventeen studies (11.8 %) adopted a blinded design.

The main experimental treatment investigated in these studies was RT alone or combined with systemic treatments (chemotherapy/target therapy) (n = 59, 37.4 %), followed by targeted therapy (n = 52, 33 %), immunotherapy alone or associated with chemotherapy (n = 18, 11.4 %), chemotherapy alone (as first, second, third line treatment and beyond) (n = 23, 14.5 %) and other treatments (surgery as single modality treatment, proton therapy) (6, 3.7 %). Concerning the HNC setting, 78 studies (49.4 %) involved recurrent/metastatic setting, while the exclusive treatment, represented by RT combined with chemotherapy or surgery, is found in 39 articles (24.7 %), induction/neoadjuvant treatment in 26 (16.4 %), the adjuvant treatment in 12 (7.6 %) and other treatment modalities in 3 (1.9 %).

### 3.2 Inclusion of QoL among study endpoints

**Fig. 2:**
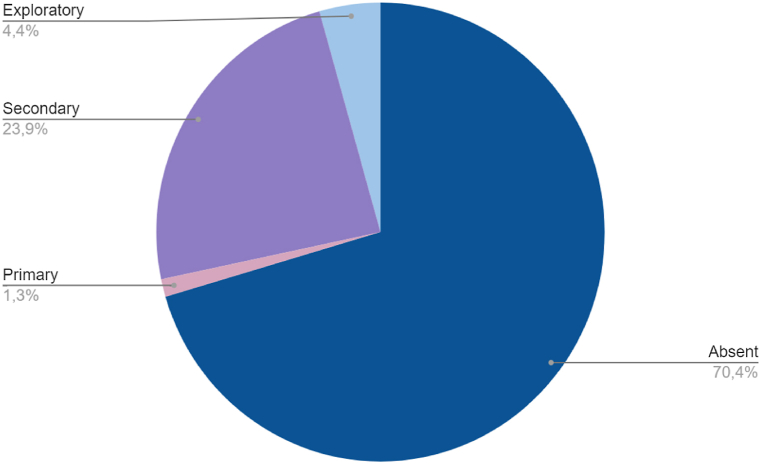
Inclusion of QoL among endpoints (all studies).

**Fig. 3:**
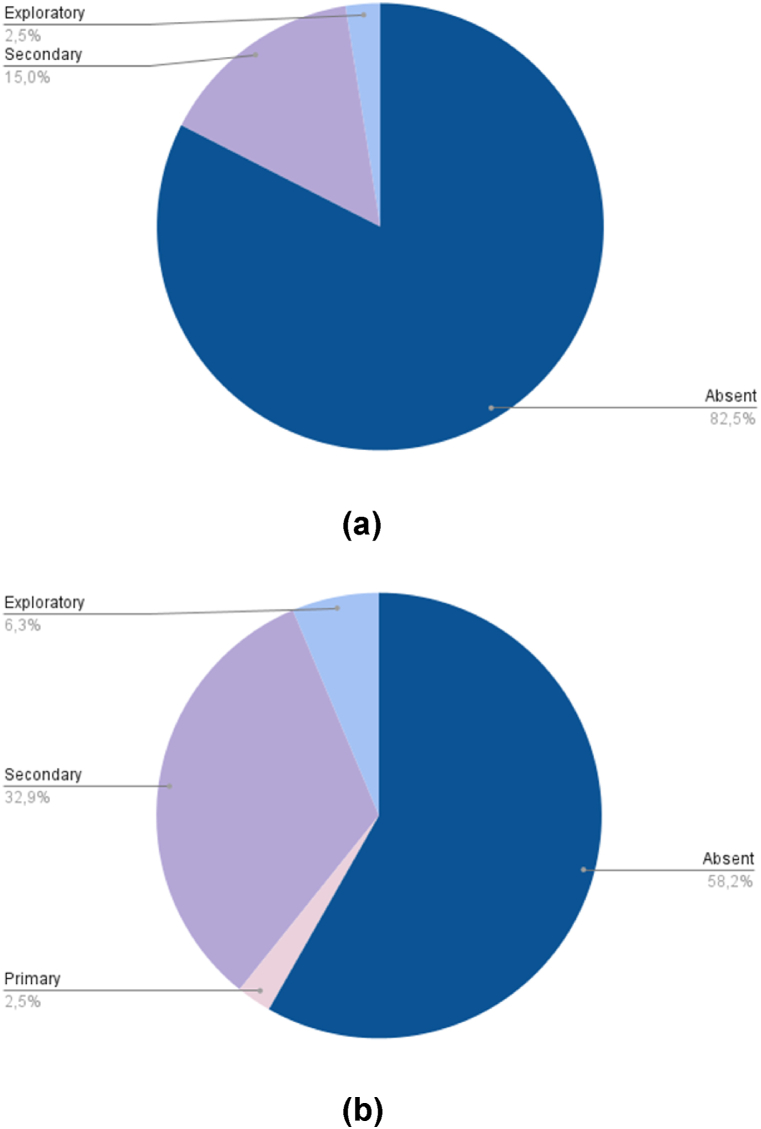
QoL results in primary publications over time. (a) QoL results in primary publications from 2008 to 2015; (b) QoL results in primary publications from 2016 to 2023.

**Fig. 4:**
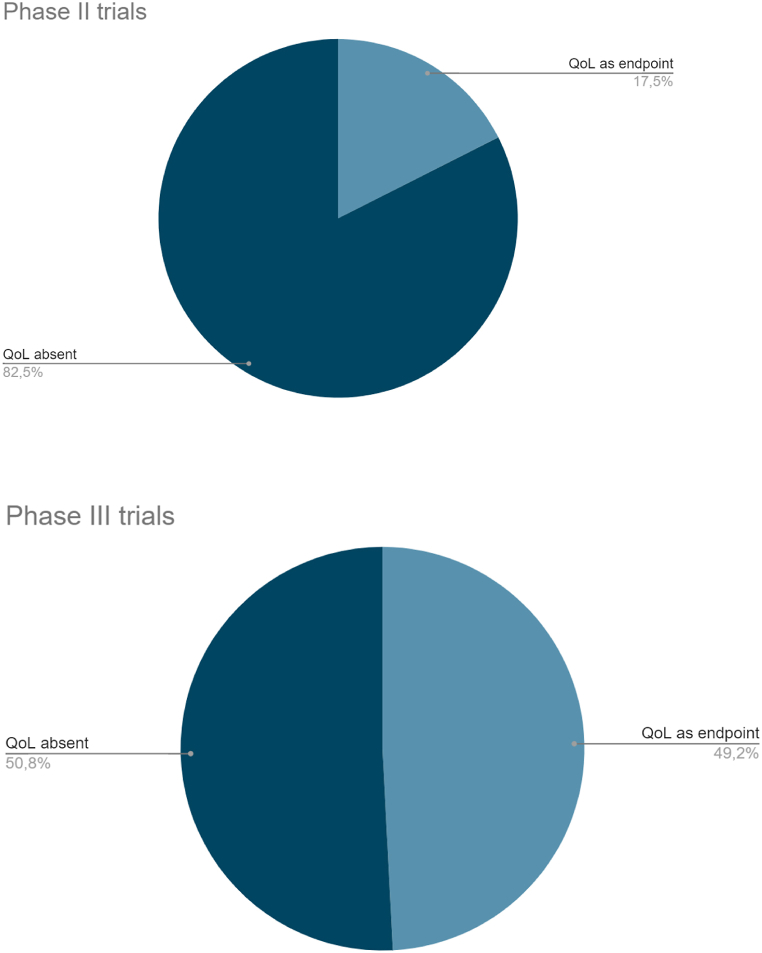
Distribution of QoL as endpoint in phase II and phase III trials. (a) QoL as endpoint in phase II trials; (b) QoL as endpoint in phase III trials.

The inclusion of QoL among endpoints and the study characteristics are summarized in Table 3. Globally, QoL was included among endpoints in 47 publications (30 %): as primary endpoint in 2 publications (1 %), as secondary endpoint in 38 (24 %) and as exploratory endpoint in 7 (4 %) (Fig. 2). The quota of primary publications with QoL among endpoints increased over time: QoL was reported in 14 (17.5 %) publications between 2008 and 2015 and in 33 publications (42 %) between 2016 and 2023 [Fig. 3 (a, b)]. In phase III trials, which are likely to lead to the approval of new standards of care, QoL was included among endpoints in about half (30, 49.2 %) of the publications, while in phase II trials QoL was present in only seventeen publications (17.5 %) [Fig. 4 (a, b)].

**Table 3:** Inclusion of QoL among study endpoints according to study features.

Feature	N. of publications	QoL primary endpoint	QoL secondary endpoint	QoL exploratory endpoint	QoL not included as endpoint
Whole series	158	2 (1.2 %)	38 (24 %)	7 (4.4 %)	111 (70.4 %)
**Year of primary publication**					
2008	11	–	1 (10 %)	–	10 (90 %)
2009	2	–	–	–	1 (100 %)
2010	13	–	1 (7.6 %)	2 (15.4 %)	10 (77 %)
2011	12	–	4 (33.4 %)	–	8 (66.6 %)
2012	7	–	1 (14.3 %)	–	6 (85.7 %)
2013	13	–	–	–	13 (100 %)
2014	10	–	2 (20 %)	–	8 (80 %)
2015	12	–	3 (25 %)	–	9 (75 %)
2016	7	–	3 (42.8 %)	–	4 (57.2 %)
2017	12	–	3 (25 %)	1 (8.3 %)	8 (66.7 %)
2018	13	–	4 (30.7 %)	1 (7.7 %)	8 (61.6 %)
2019	14	1 (7.1 %)	6 (42.9 %)	1 (7.1 %)	6 (42.9 %)
2020	9	1 (11.1 %)	1 (11.1 %)	–	7 (77.8 %)
2021	10	–	4 (40 %)	–	6 (60 %)
2022	14	–	5 (35.7 %)	2 (14.3 %)	7 (50 %)
2023	0	–	–	–	–
**Journal Impact Factor**					
Low (<15)	41	–	11 (29 %)	–	29 (25.8 %)
High (>30)	117	2 (100 %)	27 (71 %)	7 (100 %)	82 (74.2 %)
**Study design**					
Phase II	97	1 (50 %)	14 (36.9 %)	2 (28.6 %)	80 (72.3 %)
Phase III	61	1 (50 %)	24 (63.1 %)	5 (71.4 %)	31 (27.7 %)
**Type of experimental therapy**					
RT ± other (chemotherapy, target therapy)	59	–	18 (47.3 %)	4 (57 %)	37 (33.3 %)
Immunotherapy alone or in combination	18	–	6 (15.8 %)	3 (43 %)	9 (8.1 %)
Chemotherapy alone	23		3 (8 %)		20 (18.1 %)
Targeted therapy	52	–	9 (23.7 %)	–	43 (38.7 %)
Others	6	2 (100 %)	2 (5.2 %)	–	2 (1.8 %)
**Treatment setting**					
Induction/Neoadjuvant	26		3 (8 %)		23 (20.7 %)
Adjuvant	12		3 (8 %)	1 (14.2 %)	8 (7.2 %)
Exclusive	39	2 (100 %)	16 (42 %)	3 (42.9 %)	18 (16.2 %)
Recurrent/metastatic	78		14 (36.8 %)	3 (42.9 %)	61 (54.9 %)
Other	3		2 (5.2 %)		1 (1 %)
**Primary endpoint**					
OS	36	–	12 (31.7 %)	4 (57.1 %)	20 (18 %)
PFS	32	–	7 (18.4 %)	1 (14.3 %)	24 (21.7 %)
ORR	54	–	7 (18.4 %)	1 (14.3 %)	46 (41.4 %)
LRC	13	–	4 (10.5 %)	–	9 (8.1 %)
Others^a^	23	2 (100 %)	8 (21 %)	1 (14.3 %)	12 (10.8 %)
**Primary endpoint result**					
Positive	92	2 (100 %)	21 (55.3 %)	4 (57.1 %)	65 (58.6 %)
Negative	66	–	17 (44.7 %)	3 (42,9 %)	46 (41.4 %)

a Including the two trials with QoL as primary endpoint.

Among treatment setting, QoL was present as endpoint mostly in the exclusive setting (n = 21, 44.7 %), followed by recurrent/metastatic setting (n = 17, 36.3 %), adjuvant (n = 4, 8.5 %), induction/neoadjuvant (n = 3, 6.3 %) and other settings (n = 2, 4.2 %).

According to the results of primary endpoints, the whole series was divided into “positive” (92, 58 %) and “negative” studies (67, 42 %). Among the 92 clinical trials with positive results, 65 (71 %) did not include QoL as an endpoint.

A subsequent exploratory analysis was conducted among secondary publications, subgroup analysis and updates of previously published clinical trials (a total of 36 studies were analyzed); in eleven of them (30 %), QoL results were reported.

### 3.3 QoL assessment

Considering the 47 trials with available QoL results, the tools adopted for QoL evaluation included both cancer-generic tools such as European Organization for Research and Treatment of Cancer Quality of Life Questionnaire–Core 30 (EORTC QLQ-C30) with the addition of utility tools as EuroQoL-5Dimension (EQ-5D), and HNC-specific tools such as EORTC QLQ-Head and Neck 35 (EORTC QLQ-HN35), Functional Assessment of Cancer Therapy-Head and Neck (FACT-HN), Functional Assessment of Cancer Therapy Head and Neck Cancer Symptom Index (FHNSI-10). Other studies involved symptoms-specific QoL tools, adapted for HNC setting, as MD Anderson Dysphagia Index (MDADI), University of Washington Quality of Life (UW-QOL), Swallowing Quality of Life (SWAL-QOL), Voice-related Quality of Life (VRQOL), Voice Handicap Index (VHI-10), Neck Dissection Impairment Index (NDII).

## 4 Discussion

This systematic review demonstrated that QoL outcomes are still underreported in phase II and phase III clinical trials evaluating patients with HNC. These trials prioritize the assessment of clinical efficacy and survival outcomes, thereby constraining the comprehensive evaluation of well-documented acute and long-term toxicities associated with the administered treatments. The need for QoL assessment was recognized by a positive trend of clinical trials reporting QoL as a study endpoint over the years. Despite the availability of specialized tools for QoL assessment, a significant number of studies still omit QoL as a trial endpoint. When QoL is included, it is often assessed using tools that are not specifically designed for HNC.

Furthermore, despite the collection of QoL data in accordance with the study protocol, the primary publication may not consistently present this essential information.

Notably, in 2009, the US Food and Drug Administration (FDA) finalized guidance for the inclusion of PROs in clinical trials [29]. In 2014, the European Medicines Agency (EMA) published a reflection paper on the inclusion of PROs in research [30], aiming at reaching a patient-centered standard of care. Nevertheless, QoL outcomes are significantly limited in the available published literature, and this deficiency extends beyond HNC.

As reported in a previous publication by Hwang et al., analyzing 352 randomized clinical trials (RCTs) for metastatic solid cancers published between 2010 and 2015, 46 % of RCTs did not include QoL among study endpoints [31]. Similarly, a review of phase 3 RCTs for solid tumors conducted from 2012 to 2016 by Marandino et al. showed that 47 % of publications missed QoL among endpoints [32].

Servetto et al., examining QoL reports in RCTs of immunotherapy in solid cancers, revealed that QoL data, despite being often assessed and included into endpoints, were not reported in 78.3 % of primary publications [33]. Regardless of a positive trend from 2018 to 2021 in reporting QoL results (8.3 %–33.3 %), these were presented in secondary publications with a relevant delay from the initial study, hindering the chance to correctly interpret the balance between clinical efficacy of a new treatment and its potential burden for patients. Another analysis of QoL assessment in clinical trials in lung cancer demonstrated a low rate of inclusion of QoL results in primary publications of RCTs, especially if published in high impact factor (IF) journals [34]. Moreover, QoL was not listed among endpoints in 32 % of the phase III RCTs on prostate cancer from 2012 to 2018 [32] and in 65 % of phase III RCTs on colorectal cancer between 2012 and 2018 [35]. However, the proportion of clinical trials including QoL among endpoints progressively increased over time, despite the suboptimal reports of QoL results in primary publications [36].

If treatments compared in RCTs demonstrate similar efficacy, PROs may be essential to choose the most appropriate type of treatment [37,38], especially in HNC setting. However, even if the superiority of a treatment is demonstrated, reporting QoL results may be useful to identify the current value of the specific treatment, allowing the clinician to have a greater awareness regarding the treatment proposal and consequently to better illustrate the risks and benefits of each treatment to the patient.

The need to understand the individual treatment-related burden of toxicities is particularly relevant in patients with HNC, who constitute a vulnerable population experiencing symptoms control difficulties and potentially severe adverse effects due to surgical and medical treatments in addition to a fragile physical, social, and psychological background. Multimodal treatments are undeniably linked to enhanced outcomes in HNC. Moreover, given the identification of HPV as an etiological factor potentially associated with a more favorable prognosis in oropharyngeal cancer, achieving higher cure rates, a careful consideration of the treatment-related morbidities that persist in long-term survivors is crucial. This has spurred a growing interest in less-intensive clinical approaches that aim to deescalate therapeutic strategies, to mitigate toxicities while optimizing oncological outcomes [39,40].

With the introduction of ICIs in HNC treatment strategies, it is important to consider the actual benefit obtained from these new high-cost drugs, which, although in a minority of patients, may lead to heavy toxicities, potentially involving any organ and persisting even after cessation of immunotherapy, requiring long-term corticosteroid treatment or hormone replacement, further associated to additional side effects [41]. Moreover, many generic questionnaires used to assess QoL outcomes as well as HNC-specific QoL tools in patients with HNC do not consider the symptoms related to adverse effects of ICIs, potentially making them less sensitive in this context.

The assessment of QoL becomes even more pertinent when the primary endpoint of a trial is a surrogate measure, distinct from overall survival (OS), such as progression-free survival (PFS) or locoregional response rate (LRR), necessitating a comprehensive evaluation from the patient's perspective.

In our analysis, QoL results in clinical trials about HNC are mostly evaluated as secondary or exploratory endpoints, lacking predefined hypothesis or subsequent confirmatory study, and often obtained by generic assessment instruments (e.g. EORTC QLQ.C30), despite the availability of specific tools designed for patients with HNC. Moreover, QoL results are frequently underreported and relegated to supplementary materials in many instances, impacting the overall validity of the data.

Among clinical trials from 2008 to 2023, the proportion of publications with QoL results has increased over time, indicating particular attention to the role of QoL outcomes in patients with HNC, especially in long-term survivors. In fact, we divided the publications into two subgroups (from 2008 to 2015 and from 2016 to 2023), demonstrating a positive trend in QoL data reports (from 17.5 % to 41.7 %).

However, a significant proportion (70.6 %) of clinical trials with a positive primary endpoint did not include QoL results in the primary publication. Because phase II and phase III clinical trials may often lead to the approval of a new drug as standard of treatment, QoL assessment should be performed and analyzed carefully before its introduction in clinical practice.

For relevant clinical trials such as the EXTREME trial, the KEYNOTE-040 trial, and those exploring de-escalation treatments for HPV-positive HNC, we have found QoL outcomes among updates or secondary analyses of prior publications.

We acknowledge the limitations of our study. First, our study is not comprehensive of the entire current literature about HNC because we analyzed phase II and phase III trials published in 11 major journals. However, following the methodology reported in a previous publication [33], we chose to include journals that are among those most read by the oncological community, considering that a large proportion of clinical trials including QoL among endpoints is frequently published in journals with higher IF. Despite this limitation, the review may offer a practical representation of clinical trials in HNC, emphasizing the necessity for a thorough QoL assessment.

Second, we did not analyze in detail the QoL assessment reported in each publication (i.e. modality of collection of data, statistical analysis used, different QoL scales reported). In the majority of cases, the QoL analysis was made during active treatments, and the study of the long-term adverse effects, which are so commonly invalidating in patients with HNC, is investigated in further secondary analysis which are not available at the moment of primary publication. To better identify the clinical needs of these patients, the results of long-term treatment-related toxicities should be more comprehensively examined and incorporated into clinical trials. This may involve the seamless collection of data through electronic instruments, such as questionnaires administered to patients over an extended period.

We aim to conduct further studies, with a comprehensive screening of all the literature available about HNC, including journals specifically covering radiotherapy and surgical treatments, in order to investigate the current perspective about QoL relevance in HNC clinical trials.

In conclusion, the main challenge is an improvement of clinical endpoints of efficacy associated with an acceptable health-related QoL in patients with HNC, considering all the limitations linked to the extremely difficult QoL assessment due to subjective perception of individual QoL, which includes symptoms, functional status, and satisfaction with care.

## 5 Conclusions

Despite the growing awareness of PROs in recent years and the availability of validated instruments specifically designed for assessing QoL in HNC, the incorporation of QoL as an endpoint in clinical trials remains conspicuously deficient. This gap in assessment is noteworthy due to the unique challenges posed by patients with HNC, necessitating a comprehensive, multidisciplinary approach and an extended follow-up strategy.

## CRediT authorship contribution statement

**Daria Maria Filippini:** Writing – review & editing, Writing – original draft, Validation, Supervision, Project administration, Methodology, Investigation, Funding acquisition, Data curation, Conceptualization. **Francesca Carosi:** Writing – review & editing, Writing – original draft, Visualization, Validation, Methodology, Investigation, Data curation, Conceptualization. **Olimpia Panepinto:** Writing – review & editing, Investigation. **Giacomo Neri:** Software, Resources, Investigation. **Elisabetta Nobili:** Supervision, Resources, Methodology, Conceptualization. **Nastassja Tober:** Visualization, Data curation. **Raffaele Giusti:** Writing – review & editing, Validation, Supervision. **Massimo Di Maio:** Writing – review & editing, Visualization, Validation, Supervision.

## Data availability

Data will be made available on request.

## Funding

The work reported in this publication was funded by the 10.13039/501100003196Italian Ministry of Health, RC-2024-2790047 project.

## Declaration of Competing Interest

The authors declare the following financial interests/personal relationships which may be considered as potential competing interests: Daria Maria Filippini reports financial support was provided by 10.13039/501100003196Italian Ministry of Health, RC-2024-2790047 project. If there are other authors, they declare that they have no known competing financial interests or personal relationships that could have appeared to influence the work reported in this paper.
