# Supplementary material for: Health-related quality of life assessment in head and neck cancer: A systematic review of phase II and III clinical trials
Source: Heliyon. 2024 Nov 24;10(23):e40671. doi: 10.1016/j.heliyon.2024.e40671 (PMC11647859; doi:10.1016/j.heliyon.2024.e40671)
Supplement: Multimedia component 1 [file mmc1.docx]

**Supplementary Materials

Table 4. Studies included and their characteristics.**

| **Author** | **Year of publication** | **Journal impact factor (1:<15, 2:15-30, 3:>30)** | **Study design (1. superiority, 2. non-inferiority, 3. phase 2)** | **Type of experimental therapy (1. RT+chemo/target 2. chemo alone 3. immunotherapy 5. target 6. other (surgery)** | **Primary endpoints (OS, PFS...)** | **Study results (1. positive, 2. negative)** | **QoL as endpoint [primary (p), secondary (s), exploratory (e), apparently absent (a)]** | **QoL results [positive (p), negative (n), no difference]** |
| --- | --- | --- | --- | --- | --- | --- | --- | --- |
| Laskar et al. | 2023 | 1 | 1 | 1 | LRC | 2 | a | - |
| Psyrri A et al. | 2022 | 3 | 1 | 3 | OS | 2 | a | - |
| Patil VM et al. | 2023 | 3 | 3 | 1 | DFS, OS | 1 | s | n |
| Patil VM el al. | 2023 | 3 | 1 | 3 | OS | 1 | s | p |
| Tao et al. | 2022 | 3 | 3 | 3 | LRC | 2 | s | no difference |
| Racadot et al. | 2023 | 1 | 1 | 5 | DFS | 2 | s | n |
| Haddad et al. | 2022 | 3 | 1 | 3 | OS | 2 | e | p |
| Miao et al. | 2022 | 3 | 1 | 1 | FFS (Failure Free Survival) | 1 | a | - |
| Kiyota et al. | 2022 | 3 | 2 | 1 | OS | 1 | a | - |
| Palma et al. | 2022 | 3 | 3 | 1 | OS | 2 | s | non valutabile |
| Chaukar et al. | 2022 | 3 | 3 | 2 | mandible preservation rate | 1 | a | - |
| Ferris et al. | 2022 | 3 | 3 | 1 | PFS | 1 | e | p |
| Locati et al. | 2021 | 3 | 3 | 5 | ORR | 1 | a | - |
| Galot et al. | 2022 | 1 | 3 | 3 | ORR | 2 | a | - |
| Guo et al. | 2022 | 1 | 1 | 5 | PFS | 1 | a | - |
| Nutting et al. | 2021 | 1 | 1 | 1 | LRC | 2 | s | n |
| Keil et al. | 2021 | 1 | 3 | 5 | ORR | 1 | a | - |
| Hasegawa et al. | 2021 | 3 | 2 | 6 | OS | 1 | s | p |
| Ho et al. | 2021 | 3 | 3 | 5 | ORR | 1 | a | - |
| Sacco et al. | 2021 | 3 | 3 | 3 | ORR | 1 | a | - |
| Lee et al. | 2021 | 3 | 1 | 3 | OS | 2 | a | - |
| Guigay et al. | 2021 | 3 | 3 | 2 | OS | 2 | s | p |
| Swiecicki et al. | 2021 | 1 | 3 | 5 | Safety and PFS | 1 | a | - |
| Gebre-Medhin et al. | 2021 | 3 | 1 | 5 | OS | 2 | s | NA |
| McBride et al. | 2021 | 3 | 1 | 3 | ORR | 2 | a | - |
| Shoenfeld et al. | 2020 | 3 | 3 | 3 | Safety | 1 | a | - |
| Sun et al. | 2020 | 3 | 3 | 1 | LRC | 1 | a | - |
| Hanna GC | 2020 | 1 | 3 | 5 | PFS | 1 | a | - |
| Seiwert et al. | 2020 | 1 | 3 | 5 | PFS | 2 | a | - |
| Ferris et al. | 2020 | 3 | 1 | 3 | OS | 2 | a | - |
| Kochanny et al. | 2020 | 1 | 3 | 5 | OS | 2 | a | - |
| Dziegielewski PT et al. | 2020 | 1 | 2 | 6 | Neck Dissection Impairment Index (NDII) score | 1 | p | p |
| Locati et al. | 2020 | 1 | 3 | 5 | ORR | 1 (modest) | s | n |
| Forster et al. | 2020 | 1 | 3 | 5 | PFS | 2 | a | - |
| Argiris et al. | 2019 | 3 | 1 | 5 | OS | 2 | a | - |
| Burtness et al. | 2019 | 3 | 1 | 3 | OS, PFS | 1 | s | p |
| Klinghammer et al. | 2019 | 1 | 3 | 2 | PFS | 2 | a | - |
| Guo et al. | 2019 | 3 | 1 | 5 | PFS | 1 | s | p |
| Chera et al | 2019 | 3 | 3 | 1 | PFS | 1 | s | p |
| Nichols et a. | 2019 | 3 | 3 | 6 | QoL | 1 | p | p |
| Zhang et al. | 2019 | 3 | 1 | 2 | OS, RFS | 1 | a | - |
| Patil et al. | 2019 | 1 | 1 | 5 | PFS | 1 | a | - |
| Adkins et al. | 2019 | 3 | 3 | 5 | ORR | 1 | a | - |
| Burtness et al. | 2019 | 3 | 1 | 5 | DFS | 2 | s | no difference |
| Haddad et al. | 2019 | 3 | 3 | 2 | PFS | 1 | a | - |
| Siu et al. | 2019 | 3 | 3 | 3 | ORR | 1 | s | p |
| Cohen et al. | 2019 | 3 | 1 | 3 | OS | 1 | e | p |
| Gillison M et al. | 2019 | 3 | 2 | 1 | OS | 2 | s | p |
| Mehanna et al. | 2019 | 3 | 1 | 1 | Toxicities | 2 | e | no difference |
| Zandberg et al. | 2019 | 1 | 3 | 3 | ORR | 1 | s | p |
| Nutting et al. | 2018 | 1 | 1 | 1 | Hearing loss | 2 | s | n |
| Geoffrois et al. | 2018 | 3 | 2 | 1 | PFS | 2 | a | - |
| Ferris et al. | 2018 | 3 | 1 | 3 | PFS | 2 | a | - |
| Dietz et al. | 2018 | 3 | 1 | 5 | Laringectomy free survival | 1 | a | - |
| Weiss JM et al. 2018 | 2018 | 1 | 3 | 1 | ORR | 1 | s | p |
| Noronha et al. | 2018 | 3 | 2 | 1 | LRC | 2 | s | NA |
| Tahara et al. | 2018 | 3 | 3 | 2 | ORR | 1 | a | - |
| Awan et al. | 2018 | 3 | 3 | 1 | PFS | 1 | a | - |
| Machiels et al. | 2018 | 3 | 3 | 5 | Metabolic Response | 1 | a | - |
| Fushimi et al. | 2018 | 3 | 3 | 5 | PFS | 1 | a | - |
| Drilon et al. | 2018 | 3 | 3 | 5 | ORR | 1 | a | - |
| Plaschke et al. | 2018 | 1 | 3 | 6 | Local tumor response | 1 | s | p |
| Bossi et al. | 2017 | 3 | 3 | 2 | PFS | 1 | a | - |
| Dunn et al. | 2017 | 3 | 3 | 2 | ORR | 1 | a | - |
| Ghi et al. | 2017 | 3 | 1 | 1 | OS | 1 | a | - |
| Specenier et al. | 2017 | 3 | 2 | 1 | Feasibility | 2 | a | - |
| Harrington et al. | 2017 | 3 | 2 | 3 | OS | 1 | e | p |
| Inhestern et al. | 2017 | 3 | 3 | 2 | PFS | 1 | s | No difference (apart for social eating) |
| Chen et al. | 2017 | 3 | 3 | 1 | PFS | 1 | s | p |
| Kim et al. | 2017 | 1 | 3 | 5 | RR | 2 | a | - |
| Bauml et al. | 2017 | 3 | 3 | 3 | ORR | 1 | a | - |
| Soulières et al. | 2017 | 3 | 3 | 5 | PFS | 1 | a | - |
| Siu et al. | 2017 | 3 | 2 | 1 | PFS | 2 | s | No difference |
| Locati et al. | 2016 | 1 | 3 | 5 | RR | 1 | a | - |
| Ferris et al. | 2016 | 3 | 3 | 1 | PFS | 1 | a | - |
| Jimeno et al. | 2016 | 1 | 3 | 5 | ORR | 2 | a | - |
| Ferris et al. | 2016 | 3 | 1 | 3 | OS | 1 | s | p |
| Argiris et al. | 2016 | 3 | 1 | 1 | PFS | 1 (cetuximab), 2 (bevacizumab) | s | n |
| Villaflor et al. | 2016 | 3 | 3 | 2 | Change tumor size | 2 | a | - |
| Magrini et al. | 2016 | 3 | 3 | 1 | Compliance to treatment | 2 | a | - |
| Wong et al. | 2016 | 3 | 3 | 5 | RR | 2 | a | - |
| Driessen et al. | 2016 | 1 | 3 | 1 | Feasibility | 2 | s | n |
| Harrington et al. | 2015 | 3 | 1 | 1 | DFS | 2 | s | n |
| Guigay et al. | 2015 | 3 | 2 | 2 | ORR | 1 | a | - |
| Jensen et al. | 2015 | 1 | 1 | 6 | LCR,PFS,OS | 1 | a | - |
| D'Cruz et al. | 2015 | 3 | 1 | 6 | OS | 1 | a | - |
| Keam et al. | 2015 | 1 | 3 | 5 | PFS | 1 | a | - |
| Massarelli et al. | 2015 | 3 | 3 | 5 | ORR | 2 | a | - |
| Machiels et al. | 2015 | 3 | 1 | 5 | PFS | 1 | s | p |
| Melichar et al. | 2015 | 3 | 3 | 5 | Objective response | 1 | a | - |
| Grunwald et al. | 2015 | 3 | 3 | 2 | PFR | 1 | a | - |
| Jimeno et al. | 2015 | 3 | 3 | 5 | PFS | 1 | a | - |
| Mesia et al. | 2015 | 3 | 3 | 1 | Local regional control | 2 | a | - |
| GIralt et al. | 2015 | 3 | 3 | 1 | Local regional control | 2 | a | - |
| Miah et al. | 2015 | 1 | 3 | 1 | Xerostomia | 1 | a | - |
| Wanebo et al. | 2014 | 3 | 3 | 2 | EFS | 1 | a | - |
| Ang et al. | 2014 | 3 | 1 | 1 | LRF | 1 | s | NA |
| Cohen et al. | 2014 | 3 | 1 | 1 | OS | 2 | a | - |
| Seiwert et al. | 2014 | 3 | 1 | 5 | tumor shrinkage | 1 | s | p |
| Harari et al. | 2014 | 3 | 3 | 1 | DFS | 1 | a | - |
| Vermorken et al. | 2014 | 3 | 3 | 5 | PFS | 2 | a | - |
| Hitt et al. | 2014 | 3 | 1 | 1 | PFS, TTF | 2 | a | - |
| Vermorken et al. | 2013 | 1 | 3 | 5 | PFS | 2 | a | - |
| Schmitz et al. | 2013 | 3 | 2 | 5 | Safety | 1 | a | - |
| Vermorken et al. | 2013 | 3 | 1 | 5 | OS | 2 | a | - |
| Harrington et al. | 2013 | 1 | 3 | 1 | CRR | 1 | a | - |
| Argiris et al. | 2013 | 3 | 1 | 5 | OS | 2 | a | - |
| Martins et al. | 2013 | 3 | 1 | 5 | CRR | 2 | a | - |
| Haddad et al. | 2013 | 3 | 1 | 1 | OS | 2 | a | - |
| Lefebvre et al. | 2013 | 3 | 3 | 1 | rate of laryngeal preservation 3 months post treatment | 2 | a | - |
| Abdul Razak et al. | 2013 | 3 | 3 | 5 | ORR | 1 | a | - |
| Adkins D et al. | 2013 | 1 | 3 | 1 | CR after 2 cycles | 1 | a | - |
| Mesia R et al. | 2013 | 3 | 3 | 1 | LRC | 2 | a | - |
| Keil F et al. | 2013 | 1 | 3 | 1 | LRC | 1 | a | - |
| Fury MG et al. | 2012 | 1 | 3 | 1 | PFS | 1 | a | - |
| Urba s et al. | 2012 | 1 | 1 | 2 | OS | 2 | s | no differences |
| Schmitz S et al. | 2012 | 3 | 3 | 5 | DCR | 2 | a | - |
| Hitt R et al. | 2012 | 3 | 3 | 5 | RR | 1 | a | - |
| Gilbert J et al. | 2012 | 1 | 3 | 2 | ORR | 1 | a | - |
| Bourhis J et al. | 2012 | 3 | 2 | 1 | EFS | 2 | a |  |
| Villaflor VM et al. | 2011 | 3 | 3 | 1 | OS | 1 | a | - |
| Salama JK et al. | 2011 | 3 | 3 | 1 | PFS | 2 | a | - |
| Del Campo JM et al. | 2011 | 1 | 3 | 5 | apoptotic index | 1 | a | - |
| Boscolo-Rizzo P et al. | 2011 | 3 | 1 | 1 | LRF | 2 | s | p |
| Argiris A et al. | 2011 | 1 | 3 | 5 | ORR | 1 | a |  |
| Brooks HD et al. | 2012 | 1 | 3 | 5 | PFS, ORR | 2 | a |  |
| Machiels JP et al. | 2011 | 3 | 1 | 5 | OS | 1 | s | p |
| Argiris A et al. | 2011 | 3 | 3 | 5 | TTP | 1 | a | - |
| Merlano M et al. | 2011 | 3 | 3 | 1 | CR rate | 1 | a | - |
| Fury MG et al. | 2011 | 1 | 3 | 2 | ORR | 1 | a | - |
| Nutting CM et al. | 2011 | 3 | 1 | 1 | proportion of patients with grade 2 or worse xerostomia at 12 months, as assessed by the Late Effects of Normal Tissue (LENT SOMA) scale | 1 | s | p |
| Won YW et al. | 2011 | 3 | 3 | 2 | ORR | 1 | a | - |
| Kao J et al. | 2011 | 1 | 3 | 1 | LRR e OS | 1 | s | n |
| Argiris A et al. | 2010 | 3 | 3 | 1 | ORR | 1 | e | n |
| Sharma A et al. | 2010 | 3 | 3 | 1 | OS | 1 | a | - |
| Cohen EE et al. | 2010 | 3 | 3 | 1 | CR rate | 1 | a | - |
| Williamson SK et al. | 2010 | 3 | 3 | 5 | RR | 1 | a | - |
| Paccagnella A et al. | 2010 | 3 | 3 | 1 | Rate of CR at 6-8 weeks | 1 | a | - |
| Rischin D et al. | 2010 | 3 | 1 | 1 | OS | 2 | e | no difference |
| Martinez-Trufero J et al. | 2010 | 1 | 3 | 2 | ORR | 1 | a | - |
| Overgaard J et al. | 2010 | 3 | 1 | 1 | LRC | 1 | a | - |
| Rasch CR et al. | 2010 | 1 | 1 | 1 | LRC | 2 | s | no difference |
| C. H. Chung et al. | 2010 | 3 | 3 | 5 | OS | 2 | a | - |
| Machiels et al. | 2010 | 3 | 3 | 5 | DCR | 2 | a | - |
| Kies et al. | 2010 | 3 | 3 | 5 | ORR | 1 | a | - |
| Rosenthal et al. | 2010 | 3 | 3 | 1 | safety | 1 | a | - |
| Argiris et al. | 2009 | 3 | 3 | 2 | PFS | 2 | a | - |
| Nutting et al. | 2009 | 3 | 3 | 2 | OS | 2 | a | - |
| Franchin et al. | 2009 | 3 | 3 | 1 | compliance, OS, DFS | 1 | a | - |
| Hainsworth et al. | 2009 | 3 | 3 | 1 | Feasibility | 2 | a | - |
| Simon et al. | 2009 | 3 | 1 | 5 | OS | 2 | s | n |
| Lefebvre et al. | 2009 | 3 | 2 | 1 | OS, PFS | 2 | a | - |
| Janot et al. | 2008 | 3 | 1 | 1 | OS, LRC | 2 | a | - |
| van Herpen et al. | 2008 | 3 | 3 | 2 | Efficacy | 2 | a | - |
| Salama et al. | 2008 | 3 | 3 | 1 | RR and OS | 1 | a | - |
| Vermorken et al. | 2008 | 3 | 1 | 5 | OS | 1 | a | - |
| Gilbert et al. | 2008 | 1 | 3 | 2 | ORR | 2 | a | - |
| Burtness et al. | 2008 | 3 | 3 | 2 | RR | 2 | a | - |

LRC: locoregional control; OS: overall survival; DFS: disease free survival; PFS: progression free survival; ORR: objective response rate; RR: response rate; EFS: event free survival; RFS: recurrence free survival; NA: not available.
